# VEGFR2 inhibition hampers breast cancer cell proliferation *via* enhanced mitochondrial biogenesis

**DOI:** 10.20892/j.issn.2095-3941.2020.0151

**Published:** 2021-02-15

**Authors:** Hao Ni, Min Guo, Xuepei Zhang, Lei Jiang, Shuai Tan, Juan Yuan, HuanhuanL Cui, Yanan Min, Junhao Zhang, Susanne Schlisio, Chunhong Ma, Wangjun Liao, Monica Nister, Chunlin Chen, Shuijie Li, Nailin Li

**Affiliations:** 1 Department of Gynaecology and Obstetrics, Nanfang Hospital, Southern Medical University, Guangzhou 510515, China; 2 Karolinska Institutet, Department of Medicine-Solna, Clinical Pharmacology Group, Karolinska University Hospital-Solna, Stockholm 17176, Sweden; 3 Karolinska Institutet, Department of Oncology-Pathology, BioClinicum, Solna 17164, Sweden; 4 State Key Laboratory of Bioorganic and Natural Products Chemistry, Center for Excellence in Molecular Synthesis, Shanghai Institute of Organic Chemistry, Chinese Academy of Sciences, Shanghai 200032, China; 5 Department of Pathology, Zhejiang Provincial Key Laboratory of Pathophysiology, Ningbo University School of Medicine, Ningbo 315211, China; 6 Department of Cell and Molecular Biology, Stockholm 17177, Sweden; 7 Department of Hematology, Affiliated Hospital of Jining Medical University, Jining 272000, China; 8 Department of Oncology, Nanfang Hospital, Southern Medical University, Guangzhou 510515, China; 9 Karolinska Institutet, Department of Microbiology, Tumor and Cell Biology, Stockholm 17177, Sweden; 10 Shandong University Cheeloo Medical College, School of Basic Medicine, Department of Immunology, Jinan 250000, China

**Keywords:** Breast cancer, VEGF, VEGFR2, mitochondria, TFAM, ROS, apoptosis

## Abstract

**Objective:**

Vascular endothelial growth factor (VEGF), apart from its predominant roles in angiogenesis, can enhance cancer cell proliferation, but its mechanisms remain elusive. The purpose of the present study was therefore to identify how VEGF regulates cancer cell proliferation.

**Methods:**

VEGF effects on cancer cell proliferation were investigated with the VEGF receptor 2 inhibitor, Ki8751, and the breast cancer cell lines, MCF-7 and MDA-MB-231, using flow cytometry, mass spectrometry, immunoblotting, and confocal microscopy. Data were analyzed using one-way analysis of variance followed by Tukey’s multiple comparison test.

**Results:**

VEGF blockade by Ki8751 significantly reduced cancer cell proliferation, and enhanced breast cancer cell apoptosis. Mass spectrometric analyses revealed that Ki8751 treatment significantly upregulated the expression of mitochondrial proteins, suggesting the involvement of mitochondrial biogenesis. Confocal microscopy and flow cytometric analyses showed that Ki8751 treatment robustly increased the mitochondrial masses of both cancer cells, induced endomitosis, and arrested cancer cells in the high aneuploid phase. VEGFR2 knockdown by shRNAs showed similar effects to those of Ki8751, confirming the specificity of Ki8751 treatment. Enhanced mitochondrial biogenesis increased mitochondrial oxidative phosphorylation and stimulated reactive oxygen species (ROS) production, which induced cancer cell apoptosis. Furthermore, Ki8751 treatment downregulated the phosphorylation of Akt and PGC1α, and translocated PGC1α into the nucleus. The PGC1α alterations increased mitochondrial transcription factor A (TFAM) expression and subsequently increased mitochondrial biogenesis.

**Conclusions:**

VEGF enhances cancer cell proliferation by decreasing Akt-PGC1α-TFAM signaling-mediated mitochondrial biogenesis, ROS production, and cell apoptosis. These findings suggested the anticancer potential of Ki8751 *via* increased mitochondrial biogenesis and ROS production.

## Introduction

Breast cancer is the most common cancer in women worldwide, with more than 2,000,000 cases diagnosed, and accounts for approximately 25% of the total cancers diagnosed in 2018, according to a report of the World Cancer Research Fund (https://www.wcrf.org/sites/default/files/Breast-cancer-report.pdf). Breast cancer results in the deaths of approximately 500,000 lives each year^1^, and is the leading cause of cancer-related mortality among women, especially in women aged 20–59 years^2^. Although various types of treatments have been clinically used, the present mortality rates of breast cancer remain high, especially in those patients with triple-negative breast cancer (TNBC)^3^. Hence, there is currently intensive research to develop novel and more efficient therapies of breast cancer, which will only be achieved by our better understanding of breast cancer pathophysiology.

Platelets primarily function as thrombocytes, but are also closely involved in cancer growth and metastasis^4^. Among others, platelet-dependent angiogenic activities have emerged as an important player in the cancer microenvironment. Other studies and our laboratory studies have shown that platelets store pro- and anti-angiogenic factors in separate α-granules, and that platelets selectively release pro- and anti-angiogenic factors upon different stimuli^5,6^. Moreover, selective release of platelet angiogenic factors has been shown to have distinct impacts on angiogenesis and tumor growth^7,8^. Our recent report showed that platelet-released vascular endothelial growth factor (VEGF) is not only a key factor in platelet-enhanced tumor angiogenesis, but is also a potent promoter of cancer cell proliferation^9^. Our observations were consistent with earlier reports that VEGF is critical in maintaining cell proliferative potential, and that VEGF receptor (VEGFR) blockade by its selective inhibitor, Ki8751, significantly increases cellular senescence in metastatic colorectal cancer cells^10^. Furthermore, Ki8751 inhibition significantly delays M phase progression, and suppresses cell proliferation of HeLa S3 cells^11^.

Energy metabolism is critical for cell survival and proliferation. Normal cells conduct energy metabolism primarily through efficient mitochondrial oxidative phosphorylation (mtOXPHOS). In contrast, cancer cells produce most of their energy (approximately 60%) from aerobic glycolysis *via* the “Warburg effect,” rather than from mtOXPHOS (approximately 40%)^12^. Increasing evidence suggests that the preference of aerobic glycolysis or mtOXPHOS by cancer cells have major impacts on cancer cell biology. Thus, aggressive triple-negative and Her2^+^ breast cancer cells are more dependent on anaerobic glycolysis^13^. Luminal breast cancer cells require efficient mitochondrial respiration to maintain their tumorigenic potential^14^. Moreover, elevated levels of reactive oxygen species (ROS) can activate mtOXPHOS, and thereby increase hypoxia-inducible factor-1alpha (HIF-1α) expression to maintain the status of stem cell-like cancer cells^15^. In the present study, we aimed to identify how VEGF regulates cancer cell proliferation and how VEGF influences mitochondrial metabolism by using the VEGFR2-specific inhibitor, Ki8751. We found that VEGFR2 blockade inhibited cancer cell proliferation of the two breast cancer cell lines, MCF-7 and MDA-MB-231, and that the inhibition was achieved by enhancing mitochondrial transcription factor A (TFAM) expression and mitochondrial biogenesis. The latter led to a higher production of ROS that promoted apoptosis and subsequently hampered cancer cell proliferation.

## Materials and methods

### Culture of cancer cells

Human breast cancer cell lines, MCF-7 and MDA-MB-231, were purchased from the American Type Culture Collection (ATCC; Wesel, Germany). Both cell lines were authenticated through polymorphic short tandem repeat testing at the Uppsala Genome Center (Uppsala, Sweden). Cells between the third and tenth passages were used in the present study. The cancer cells were cultured using Dulbecco’s Modified Eagle Medium (DMEM; with high glucose, at 4.5 g/L; Thermo Fisher Scientific, Waltham, MA, USA) containing heat-inactivated 10% fetal bovine serum (FBS) and 1% penicillin-streptomycin at 37 °C with 5% CO_2_. Briefly, the MCF-7 and MDA-MB-231 cells were seeded in 6-well plates at a density of 1.5 × 10^5^ cells/well, followed by overnight attachment. The culture medium was subsequently replaced by DMEM containing vehicle (0.01% dimethyl sulfoxide) or Ki8751 (a VEGFR2-selective inhibitor; Tocris Bioscience, Bristol, UK). The cancer cells were cultured for 24, 48, or 72 h before being harvested for experiments.

### Cell proliferation assay

The MCF-7 and MDA-MB-231 cells (1.5 × 10^3^ cells in 100 μL/well) were cultured in a 96-well plate in triplicate with complete medium containing 10% FBS. Cell proliferation was determined using a cell counting kit (CCK)-8 assay (Dojindo Molecular Technologies; Rockville, MD, USA) after 0, 24, 48 and 72 h of incubation, and the absorbance was measured at 450 nm using a microplate reader^9^.

### Mass spectrometry analysis

MCF-7 breast cancer cells were cultured in the presence of 0, 2.5, and 5 μM Ki8751 for 24 h. Whole cell lysates were prepared with Laemmli buffer (Thermo Fisher Scientific) containing 10 mM dithiothreitol and 5% 2-mercaptoethanol. The procedures for mass spectrometric sample preparation have been detailed previously^16^. Briefly, after routine protein preparations, i.e., reduction, alkylation, precipitation, and digestion, the samples were desalted and fractionated to obtain 12 fractions of peptide samples. The samples were then analyzed by shotgun proteomics using nanoLC-MS/MS with a UltiMate 3000 nanoLC system and an Orbitrap Elite mass spectrometer (Thermo Fisher Scientific).

MaxQuant software (version 1.5.6.5) was used to analyze the mass spectrometric raw data and a false discovery rate (FDR) of 0.01 for proteins and peptides and a minimum peptide length of 6 amino acids were required. Only proteins quantified with at least 2 peptides were considered for quantitation, and all known contaminants were ignored. The option “Match between the runs” was used in the experiment. For each set of TMT-10-labeled samples, MS/MS reporter abundances in each channel were normalized to that of the TMT-126 channel. Principal component analysis and Orthogonal Projections to Latent Structures Discriminant Analysis (OPLS-DA) were performed using SIMCA 15.0 (Umetrics, Umea, Sweden). Normalized TMT-10 channel abundances were analyzed. OPLS-DA model performance was reported as cumulative correlation coefficients for the model [*R*^2^ × (cum)], with predictive performance being based on sevenfold cross-validation calculations [*Q*^2^(cum)]. Protein mass fold changes of 1.5 and −1.5 were set as the thresholds of upregulation and downregulation, respectively. Functional clustering of protein interactions were analyzed using the online database String (https://string-db.org/).

### Flow cytometric analyses

Cultured cancer cells were harvested using trypsin-EDTA solution, and washed twice with cold Dulbecco’s phosphate buffered saline (DPBS). For the analysis of cell apoptosis, the cells were stained with annexin V and propidium iodide (PI) using a commercial kit (Invitrogen: Carlsbad, CA, USA) according to the manufacturer’s protocol. For mitochondrial mass measurements, cancer cells were stained with 25 nM MitoTracker® Deep Red FM (Thermo Fisher Scientific) for 20 min at 37 °C in the dark. For the analysis of cell ROS, cells were stained with 2′,7′-dichlorofluorescin diacetate (20 μg/mL; Sigma-Aldrich, St. Louis, MO, USA) at 37 °C in the dark for 20 min. After a thorough washing with DPBS, the cancer cells were analyzed using an FC500 flow cytometer (Beckman Coulter; Hialeah, FL, USA).

### Western blot

MCF-7 and MDA-MB-231 cells were cultured in duplicate in 6-well plates. At various time points, cultured cancer cells were harvested and washed, and the whole cell lysates were prepared using EBC buffer (50 mM Tris at pH8.0, 120 mM NaCl, 0.5% NP-40) containing protease inhibitors (Roche Life Science, Penzberg, Germany) and phosphatase inhibitors (Sigma-Aldrich). Samples containing the same protein amounts were separated by 10% SDS-PAGE and blotted onto a nitrocellulose membrane. The nitrocellulose membranes were incubated with TBST blocking buffer (Tris-buffered saline: 25 mM Tris, 0.1% Tween 20, and 3% nonfat milk powder) for 1 h at room temperature, and further incubated at 4 °C overnight with the primary antibodies in blocking buffer. After 3–10 min washes with TBST at room temperature, the membranes were incubated with secondary antibodies in blocking buffer at room temperature for 1 h. After another 3–10 min washes in TBST, detection of immune blotting was performed using an enhanced chemiluminescence kit (ECL Plus; GE Healthcare, Chicago, IL, USA). The blotting images were analyzed using ImageJ (National Institutes of Health, Bethesda, MD, USA).

### Confocal microscopy

The cancer cells were cultured on glass coverslips, and at various time points, they were rinsed and stained with 200 nM MitoTracker Red CMXRos (Invitrogen) at 37 °C for 30 min. After staining, the cells were washed twice with prewarmed phosphate-buffered saline (PBS), and then fixed for 10 min in prewarmed 4% paraformaldehyde. Afterwards, the coverslips were incubated with PBS overnight, and then mounted using the ProLong Diamond Antifade Mountant containing 4′6′-diamidino-2-phenylindole (DAPI) (Thermo Fisher Scientific) for nuclear staining at 22 °C for 30 min. Fluorescent images were acquired with a Leica TCS SP2 AOBS (Acoustico Optical Beam Splitter) inverted laser scanning confocal microscope equipped with a 63× water immersion objective (HCX PL APO 63.0 × 1.20 water corrected; Leica, Wetzlar, Germany). The laser excitation was 415–500 nm for DAPI and 579 nm for MitoTracker Red CMXRos. The confocal pinhole (airy units) was 1 for all channels. Cell images were presented using ImageJ software.

### Cancer cell cycle analyses

MCF-7 and MDA-MB-231 cells were seeded into 12-well plates with 5 × 10^4^ cells/well, followed by adding different concentrations of Ki8751 (0, 2.5, and 5 μM) on the second day with triplicates for each concentration. After treatment for 24 h, 48 h, and 72 h, the cells were harvested using trypsin-EDTA solution, washed in PBS twice, fixed in 1 mL ice-cold 70% ethanol, and kept at −20 °C. At the time of analysis, the cells were centrifuged at 2,000 rpm for 5 min, washed with PBS twice, and then stained with 300 μL PI/RNase staining buffer (BD Pharmingen, San Jose, CA, USA) for 15 min at room temperature before analysis. Cell cycle distribution was determined using a NovoCyte flow cytometer (ACEA Bioscience; San Diego, CA, USA).

### VEGFR2, PGC1*α*, and mitochondrial transcription factor A (TFAM) knockdown of breast cancer cells

Lentivirus expressing shRNAs targeting VEGFR2 was generated in 293FT cells using pLKO.1 plasmids purchased from Sigma-Aldrich with the following sequences: shRNA-Ctrl: 5′-CCGGCAACAAGATGAAGAGCACCAACTCGAGTTGGTGCTCTTCATCTTGTTGTTTTT-3′; human VEGFR2 #86: 5′-CCGGAGGCTAATACAACTCTTCAAACTCGAGTTTGAAGAGTTGTATTAGCCTTTTTT-3′; human VEGFR2 #87: 5′-CCGGGTGCTGTTTCTGACTCCTAATCTCGAGATTAGGAGTCAGAAACAGCACTTTTT-3′; human VEGFR2 #88: 5′-CCGGCTTTACTATTCCCAGCTACATCTCGAGATGTAGCTGGGAATAGTAAAGTTTTT-3′.

MDA-MB-231 and MCF-7 cells were infected with lentivirus for 24 h. The cells were then selected for 7 days using puromycin (2 μg/mL). Afterwards, the cancer cells were harvested for Western blot for VEGFR2 expression and flow cytometric analysis of mitochondrial masses.

For PGC1α and TFAM knockdowns, the following siRNAs were used: ON-TARGETplus Human PGC1α/PPARGC1A siRNA SMARTPool (a mixture of 4 siRNAs; final concentration of 100 nM), TFAM siRNA SMARTPool (a mixture of 4 siRNAs; final concentration of 100 nM), or corresponding ON-TARGET plus Non-targeting Control Pool (Horizon Discovery; Cambridge, UK). The siRNAs were added to the cultured MCF-7 cells for siRNA transfections for 48 h using Lipofectamine™ 2000 Transfection Reagent (Thermo Fisher Scientific). At the termination of transfection, the cancer cells were harvested for flow cytometric analysis of mitochondria.

### Real time metabolic analysis

Metabolic analysis of MCF-7 and MDA-MB-231 cells were performed using the Seahorse XFp analyzer (Agilent; North Billerica, MA, USA). The cells were seeded in XFp 8-well plates (1.8 × 10^4^/well) and incubated at 37 °C with 5% CO_2_ for 24 h. Cells were then treated with vehicle or Ki8751 for 24 or 48 h prior to the assay. For the Mito Stress assay, the cells were first washed and preincubated for 1 h with Seahorse XF DMEM medium (pH 7.4) (Agilent) supplemented with 1 mM sodium pyruvate, 10 mM glucose, and 2 mM L-glutamine (Sigma-Aldrich). The oxygen consumption rate (OCR) was analyzed at basal conditions and after sequential injections of oligomycin (1 μM), carbonyl cyanide-4-(trifluoromethoxy) phenylhydrazone (FCCP; 1 μM), and antimycin/rotenone (0.5 μM). All metabolic assays were normalized to total protein content.

### Cytoplasmic and nuclear extraction

Cytoplasmic and nuclear extractions were conducted according to the manufacturer’s instructions (Thermo Fisher Scientific). Briefly, 50 μL cell pellets were harvested and washed once with PBS. After adding 500 μL ice-cold CER I buffer, the cell pellets were vortexed for 15 s. The cells were incubated on ice for 10 min followed by adding 27.5 μL ice-cold CER II buffer, and further incubating on ice for 1 min. The samples were then centrifuged at 14,000 × *g* for 15 min at 4 °C. The supernatant (cytoplasmic extract) was transferred to a clean tube. The pellet was suspended in 250 μL ice-cold NER buffer, and incubated on ice for 40 min and vortexed for 15 s every 10 min. Afterwards, the sample was centrifuged at 14,000 × *g* for 15 min at 4 °C. The supernatant (nuclear extract) was immediately transferred to a clean tube. The protein levels were quantified using the Bradford reagent (Bio-Rad, Hercules, CA, USA) and a BioPhotometer (Eppendorf, San Diego, CA, USA). The same amounts of protein (50 μg cytoplasmic proteins and 15 μg nuclear proteins) were loaded on SDS-PAGE gels for Western blot. The following antibodies were used: anti-GAPDH (ab22555; 1:1,000; Abcam, Cambridge, UK), anti-LaminB1 (ab16048; 1:1,000: Abcam), and anti-PGC-1α mouse mAb (clone 4C1.3; ST1202; Millipore, Hayward, CA, USA).

### Statistical analysis

Data are presented as the mean ± SEM. Comparisons between treatments were analyzed by one-way analysis of variance followed by Tukey’s multiple comparison tests where appropriate using GraphPad 6 (GraphPad Software, San Diego, CA, USA). *P* < 0.05 was assumed to be statistically significant.

## Results

### VEGF receptor 2 blockade enhances apoptosis of breast cancer cells

**Figure 1:**
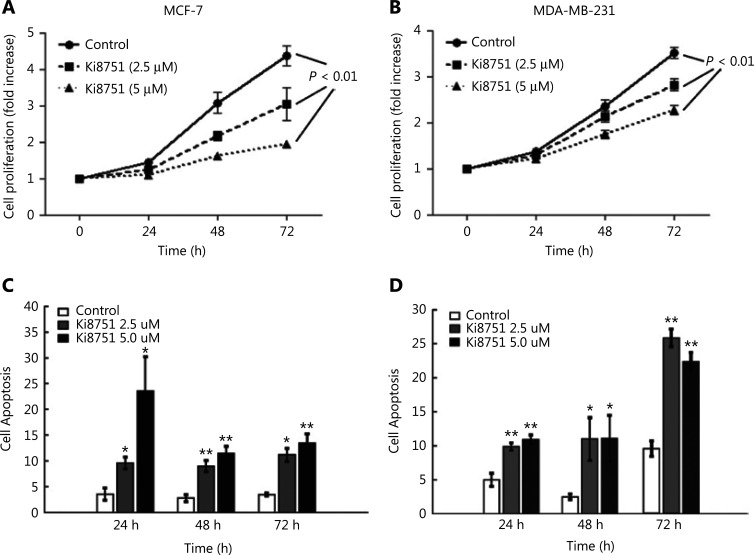
Vascular endothelial growth factor (VEGF) blockade by the VEGF receptor 2 antagonist, Ki8751m inhibits cell proliferation but enhances cell apoptosis of breast cancer cells. The breast cancer cell line, MCF-7 (panels A and C) and MDA-MB-231 cells (B and D) (1.5 × 10^3^ cells in 100 μL/well) were cultured in a 96-well plate in triplicates with complete medium (RPMI 1640 containing 10% fetal bovine serum) in the presence of vehicle (0.01% dimethyl sulfoxide), 2.5 μM, or 5 μM Ki8751 for 24, 48, and 72 h. Panels A and B: Cell proliferation was assessed using a CCK-8 assay. The fold increases of optical density values, proportional to the increase of cell numbers, were referred to those at time 0. Data are presented as the mean ± SEM; *n* = 6. *P* < 0.01, Ki8751-treated cells compared to control cells, as assessed by repeated measurements of analysis of variance. Panels C and D: Breast cancer cells were harvested and stained with fluorescein isothiocyanate-annexin V and propidium iodide. After a thorough wash, the cancer cells were analyzed using an Beckman Coulter FC500 flow cytometer. *P < 0.05; **P < 0.01, as compared to the control at corresponding time points; *n* = 4.

To illustrate the effects of VEGF on cancer cell proliferation, the breast cancer cell lines, MCF-7 and MDA-MB-231, were cultured with 10% FBS (as the source of platelet-released VEGF) in the absence or presence of Ki8751 (2.5 and 5 μM). At time points of 24, 48, and 72 h, the cell number was determined using a CCK8 assay. As shown in **Figure 1A**, MCF-7 cancer cells cultured with vehicle (control) proliferated over time, especially after 48 and 72 h when 3- to 4-fold increases of cell numbers were seen. MCF-7 cell proliferation was decreased in the presence of Ki8751 (*P* < 0.01) in a concentration-dependent manner, as compared to the control for both 2.5 μM and 5 μM Ki8751 treatments. In a similar manner, MDA-MB-231 cell proliferation was also inhibited by Ki8751 (*P* < 0.01) (**Figure 1B**).

To determine how Ki8751-mediated VEGF blockade inhibited cell proliferation, apoptosis of cultured breast cancer cells was assessed by flow cytometric analyses of annexin V- fluorescein isothiocyanate and PI staining. **Figure 1C** shows that Ki8751 treatment significantly increased apoptosis of MCF-7 cells, and that higher Ki8751 concentration was associated with a higher apoptosis rate at 24 h of culture. Ki8751 also induced apoptosis of MDA-MB-231 cells, with the highest apoptosis seen after 72 h of culture (**Figure 1D**). These findings suggested that VEGF blockade by Ki8751 triggered apoptosis of breast cancer cells. It should be noted that the same DMEM culture medium was used for both MCF-7 and MDA-MB-231 cells, to facilitate comparisons between the two cell lines. However, DMEM might not be the optimal medium for MDA-MB-231 cells. This might contribute to the delayed proliferation of MDA-MB-231 cells (**Figure 1B**), which had a shorter doubling time than MCF-7 cells^17^, and higher apoptosis after 72 h of culture (**Figure 1D**).

### VEGFR2 inhibition elevates expression of mitochondrial proteins

**Figure 2:**
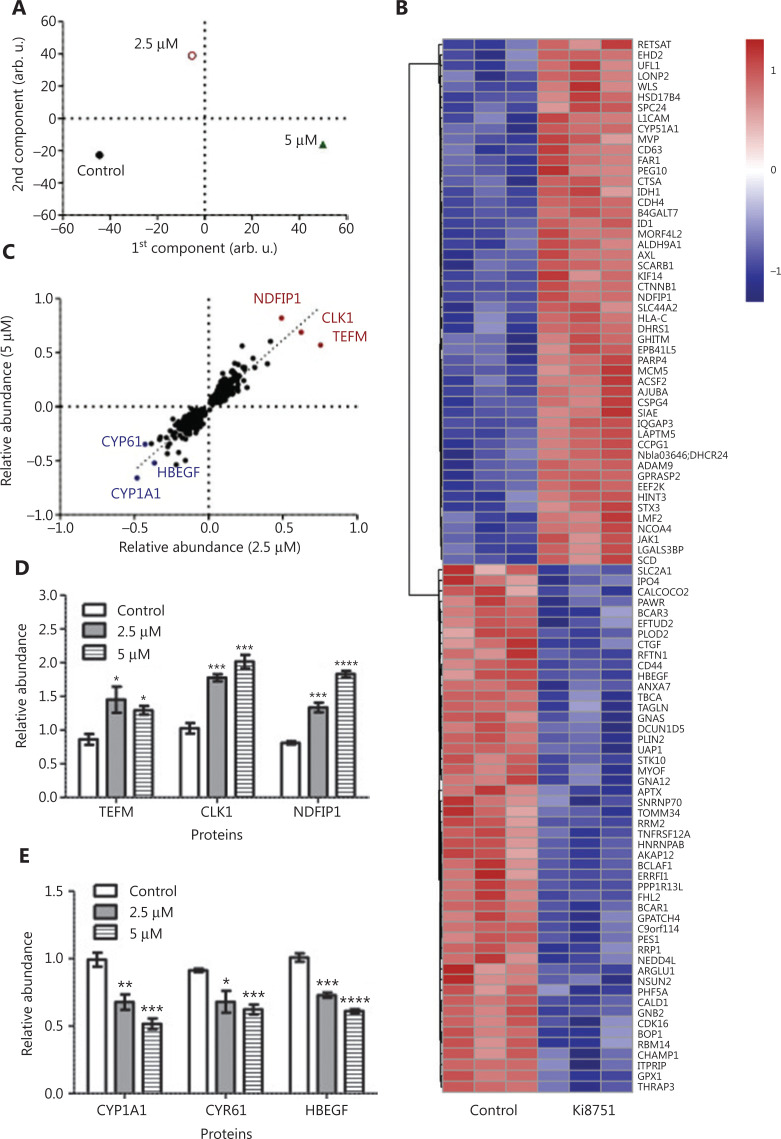
Mass spectrometric analyses highlight the upregulation of mitochondrial associated protein expression by vascular endothelial growth factor receptor (VEGFR)2 blockade. MCF-7 breast cancer cells were cultured in the presence of vehicle (0.01% dimethyl sulfoxide), 2.5, and 5 μM Ki8751 for 24 h. Whole cell lysates were prepared with Laemmli buffer containing 10 mM dithiothreitol and 5% 2-mercaptoethanol. Quantitative proteomics analyses of proteome regulation were performed using nanoLC-MS/MS among the three differently treated MCF-7 cells. Panel A: Orthogonal projections to latent structures discriminant analysis of protein abundances to reveal the differences among differently treated MCF-7 cells. B: The heat map of the top 50 up- and downregulated proteins of MCF-7 cells treated with 5 μM Ki8751. C: Correlation of significantly regulated proteins in MCF-7 cells treated with 2.5 and 5 μM Ki8751. D and E: The relative abundances of the three most up- and downregulated proteins. *P < 0.05; **P < 0.01; ***P < 0.001, as compared to the control (vehicle-treated) cells; *n* = 3.

The cellular proteomes of MCF-7 cells treated by vehicle, 2.5 μM, or 5 μM Ki8751 were extracted and analyzed by nanoLC-MS/MS. OPLS-DA (**Figure 2A**) showed a clear separation of three differently treated cells, with the replicates clustering together. A total of 7,061 proteins were identified and quantified, with 6,714 proteins common for all treatments. Among the proteins altered by Ki8751 treatments, the numbers of up- and downregulated proteins were similar. The heat map (**Figure 2B**) shows the most specifically up- and downregulated 50 proteins of 5 μM Ki8751-treated MCF-7 cells. Notably, among the proteins significantly altered by 2.5 μM and 5 μM Ki8751 treatments, 266 proteins showed a good correlation between the two treatments (R^2^ = 0.79) (**Figure 2C**). The relative abundances of the three most up- and downregulated proteins are shown in **Figure 2D and 2E**, respectively. Notably, the three most upregulated proteins, NDFIP1, CLK1, and TEFM, were mitochondrial proteins (**Figure 2C**).

### VEGFR2 inhibition increases mitochondrial mass in breast cancer cells

**Figure 3:**
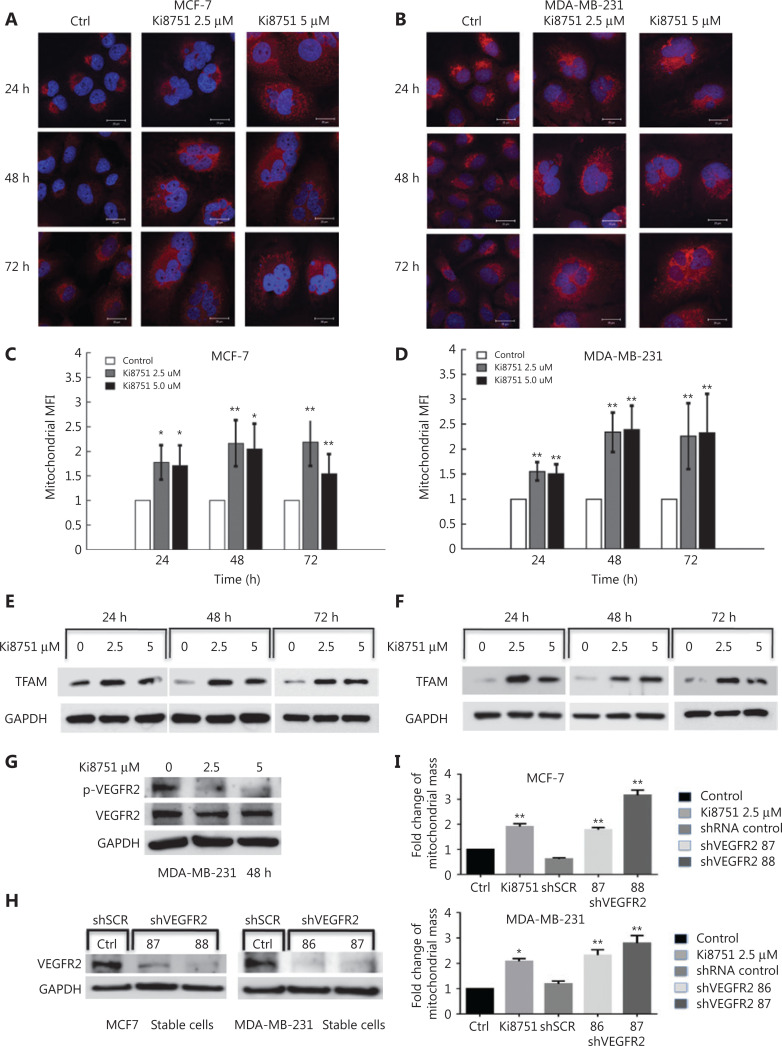
Vascular endothelial growth factor receptor (VEGFR) 2 blockade by Ki8751 and VEGFR2 knockdown increases mitochondrial mass of breast cancer cells. The breast cancer cells, MCF-7 and MDA-MB-231, were cultured in a 6-well plate and in the presence of vehicle, 2.5, and 5 μM Ki8751 for 24, 48, and 72 h. Panels A and B: The breast cancer cells were stained with MitoTracker Red CMXRos for mitochondria and with 4′6′-diamidino-2-phenylindole for nuclei. Fluorescent images were acquired with a Leica TCS SP2 AOBS (Acoustico Optical Beam Splitter) inverted laser scanning confocal microscope equipped with a 63× water immersion objective. Representative images are from not less than three independent experiments. Panels C and D: The breast cancer cells were stained with 25 nM MitoTracker^®^ Deep Red FM for 20 min at 37 °C in the dark. Mitochondrial fluorescence of the cancer cells were analyzed using a Beckman Coulter FC500 flow cytometer. *P < 0.05; **P < 0.01 as compared to the vehicle-treated controls. Panels E and F: Whole cell lysates of breast cancer cells were prepared using an EBC buffer (50 mM Tris at pH 8.0, 120 mM NaCl, 0.5% NP-40) containing protease inhibitors and phosphatase inhibitors. Samples containing the same protein amounts were separated by 10% SDS-PAGE and blotted onto nitrocellulose membranes. Western blot of mitochondrial transcription factor A (TFAM) was probed by anti-TFAM antibody, and glyceraldehyde 3-phosphate dehydrogenase (GAPDH) was used as the internal control (anti-GAPDH antibody). The results shown are representative images from 3 independent experiments. Panel G: MDA-MB-231 cells were cultured without or with Ki8751 for 48 h, and then harvested for Western blot of VEGFR2 (human VEGFR2/KDR/Flk-1 antibody) and phosphorylated VEGFR2 [phospho-VEGFR2 (Tyr951) antibody]. Panels H and I: MCF-7 and MDA-MB-231 cells were infected during 24 h with lentivirus expressing VEGFR2-targeted shRNAs at 86, 87, 88, or scrambled control. The cells were then selected for 7 days using puromycin (2 μg/mL). Afterwards, the cancer cells were harvested for Western blot of VEGFR2 expression and flow cytometric analysis of mitochondrial masses. Representative Western blot of VEGFR2 expressions in MCF-7 and MDA-MB-231 cells. (H). Fold changes of mitochondrial mass as compared to the control upon Ki8751 treatment or VEGFR knockdown are plotted (I); mean ± SEM, *n* = 3, *P < 0.05; **P < 0.01, as compared to the control.

Because the mass spectrometric analysis indicated that proteins related to mitochondrial biogenesis were upregulated by VEGFR2 blockade, we investigated mitochondrial fluorescence intensities of MCF-7 and MDA-MB-231 cells during 72 h of culture. The results showed that cell sizes of MCF-7 and MDA-MB-231 cells did not change markedly over time when cultured in the presence of vehicle (0.01% dimethyl sulfoxide) (**Figure 3A and 3B**). In contrast, when cultured in the presence of Ki8751, the cell and nuclear sizes of both MCF-7 and MDA-MB-231 cells were significantly increased, and mitochondrial staining was much brighter than that of the cells cultured with vehicle. When mitochondrial staining was analyzed by flow cytometry, VEGF blockade by Ki8751 increased mitochondrial fluorescence, and the increases of mitochondrial fluorescence were more intense at 48 h and 72 h for both cell lines (*P* < 0.01, compared to the controls), especially in MDA-MB-231 cells (**Figure 3C and 3D**).

To identify the mechanisms underlying mitochondrial biogenesis by VEGF blockade, we further investigated mitochondrial biogenesis-related signaling. TFAM is a transcription factor for mitochondrial DNA (mtDNA)^18^, and controls mitochondrial biogenesis^19^. VEGF blockade by Ki8751 increased TFAM immunoblotting intensities in MCF-7 cells at all 3 time points monitored (**Figure 3E**), indicating the elevation of TFAM expression. Similarly, Ki8751-treatment significantly enhanced TFAM expressions throughout the 3 day culture of MDA-MB-231 cells (**Figure 3F**).

### Verification of the specificity of VEGFR2 inhibition by Ki8751

To verify that the effects of Ki8751 on cancer cells were specific and dependent on VEGFR2 inhibition, a series of experiments were performed. As Ki8751 is an inhibitor of VEGFR2 phosphorylation, it was confirmed that Ki8751 treatments (both at 2.5 μM and 5 μM) reduced VEGFR2 phosphorylation levels of MDA-MB-231 cells (**Figure 3G**). Using VEGFR2-specific shRNAs, shRNA86 and shRNA87 significantly reduced VEGFR2 expression of MDA-MB-231 cells, while shRNA87 and shRNA88 significantly decreased MCF-7 cell VEGFR2 expression (**Figure 3H**). Moreover, VEGFR2-knockdown by shRNAds increased mitochondrial mass levels of MDA-MB-231 and MCF-7 cells to a similar level as Ki8751 treatment (**Figure 3I**). These results indicated that the effects of Ki8751 noted in the present study occurred *via* VEGFR2 inhibition, but not by off-target effects.

### The effects of VEGF blockade on ROS production of breast cancer cells

**Figure 4:**
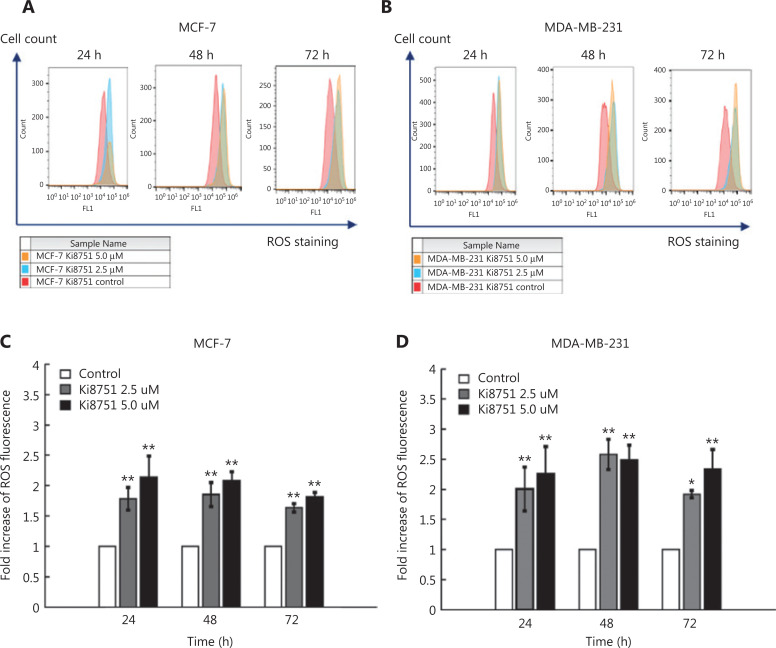
Ki8751 treatment enhances reactive oxygen species (ROS) production of breast cancer cells. The breast cancer cells, MCF-7 and MDA-MB-231, were seeded in 6-well plates at a density of 1.5 × 10^5^ cells/well with vehicle (0.01% dimethyl sulfoxide), 2.5, or 5 μM Ki8751. After 24, 48, and 72 h of culture, the cells were harvested and stained with 2′,7′-dichlorofluorescin diacetate (20 μg/mL) at 37 °C in the dark for 20 min. ROS fluorescence of the cancer cells were analyzed using a Beckman Coulter FC500 flow cytometer. Representative overlay histograms of MCF-7 (panel A) and MDA-MB-231 cells (B) were shown. Fold increases of ROS fluorescence are plotted as the mean ± SEM (MCF-7 cells, C; and MDA-MB-231 cells, D). *P < 0.05; **P < 0.01 as compared to the vehicle-treated controls; *n* = 6.

To detect the effects of VEGF blockade by Ki8751 on intracellular ROS production in MCF-7 and MDA-MB-231 cells, we monitored ROS levels using 2′,7′-dichlorodihydrofluorescein diacetate (DAPI) staining and flow cytometry^20^. **Figure 4** shows that Ki8751 treatment shifted the curves of ROS fluorescence towards the right side, i.e., intensifying ROS fluorescence for both MCF-7 cells (**Figure 4A**) and MDA-MB-231 cells (**Figure 4B**). Moreover, the increases of ROS production by Ki8751 treatment were at least > 70% (*P* < 0.01) as compared to vehicle-treated cells during the 3 day culture (**Figure 4C and 4D**). Elevation of ROS production could be higher than 2-fold, but there seemed to be no clear differences in ROS production between 2.5 μM and 5 μM Ki8751-treated cells. These results suggested that Ki8751 treatment induced a significant accumulation of ROS in breast cancer cells.

### Ki8751 treatment induces breast cancer cell cycle arrest in the high aneuploid phase

**Figure 5:**
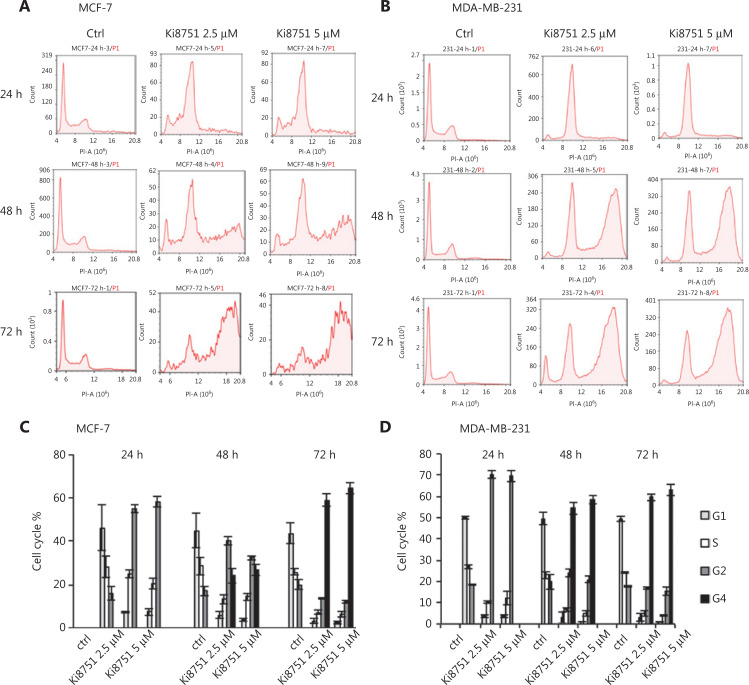
Vascular endothelial growth factor receptor (VEGFR) 2 blockade by Ki8751 arrests mitosis of breast cancer cells. The breast cancer cells, MCF-7 and MDA-MB-231, were cultured in 12-well plates at 5 × 10^4^ cells/well. After 24 h, vehicle, 2.5, or 5 μM Ki8751 were added, and the cells were further cultured for 24, 48, and 72 h. The cells were stained with propidium iodide/RNase staining buffer. DNA content of the MCF-7 cells (panel A) and MDA-MB-231 cells (B) were analyzed using a NovoCyte flow cytometer. Cell cycle analyses (C and D) were performed using the NovoExpress^®^ program.

Our confocal observations showed that Ki8751 treatment led to major morphological changes of both MCF-7 and MDA-MB-231 cells, seen as significantly enlarged cells and, most notably, cells with multiple nuclei or irregular and larger nuclei (**Figure 3A, 3B**). We therefore conducted flow cytometric cell cycle analyses to determine the effect of Ki8751 on the cell cycles of MCF-7 and MDA-MB-231 cells. **Figure 5A and 5B** shows that Ki8751 treatment, either at 2.5 μM or 5 μM, robustly increased cancer cells in the G2/M phase at 24 h, and that the treatment further increased the proportions of high aneuploid cancer cells (regarded as G4 phase) after 48 and 72 h of culture. These findings showed that Ki8751 treatments caused cell cycle arrest in the high aneuploid phase (G2/M and G4) of MCF-7 cells (**Figure 5C**) and MDA-MB-231 cells (**Figure 5D**).

### Mitochondrial biogenesis leads to increased cellular oxygen consumption

**Figure 6:**
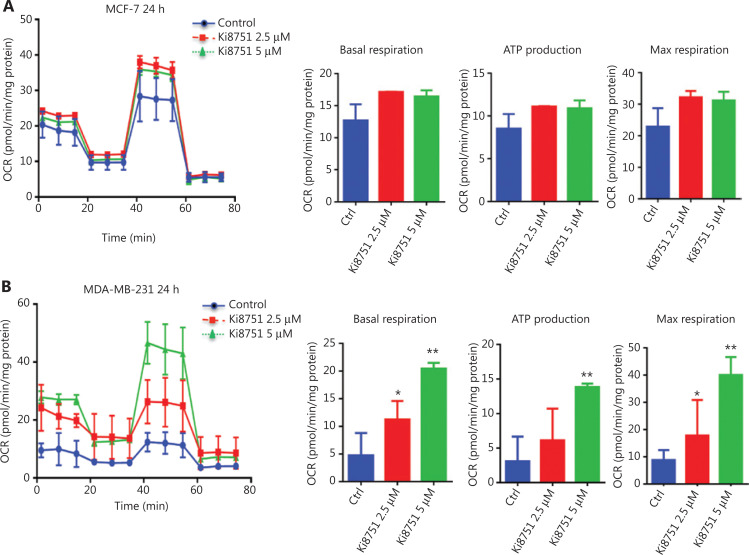
Vascular endothelial growth factor intervention increases oxygen consumption of breast cancer cells. The breast cancer cells, MCF-7 and MDA-MB-231, were cultured in XFp 96-well plates (1.8 × 10^4^/well) for 24 h. The cells were then treated with vehicle (0.01% dimethyl sulfoxide), 2.5, or 5 μM Ki8751 for 24 h prior to the assay. The oxygen consumption rate was analyzed at basal conditions and after sequential additions of oligomycin (1 μM), carbonyl cyanide-4-(trifluoromethoxy) phenylhydrazone (1 μM), and antimycin/rotenone (0.5 μM). All metabolic assays were normalized to the total protein content. Data plotted are the mean ± SEM from MCF-7 (panel A) and MDA-MB-231 cells (B). *P < 0.05; **P < 0.01, as compared to the control; *n* = 3.

VEGF blockade by Ki8751 increased mitochondrial masses of both MCF-7 and MDA-MB-231 cells. Thus, there was a need to confirm that the increase led to altered mitochondrial functions. Using the Seahorse assay, it was shown that Ki8751 treatment tended to increase the basal OCR of MCF-7 cells (**Figure 6A**), and enhanced the OCR of MDA-MB-231 cells, especially in 5 μM Ki8751-treated cells (**Figure 6B**). As expected, increases of mitochondrial masses also increased ATP production and maximal respiration levels of Ki8751-treated breast cancer cells. These enhancements were even greater in 5 μM Ki8751-treated MDA-MB-231 cells.

### The signaling cascade effect of enhanced TFAM expression

**Figure 7:**
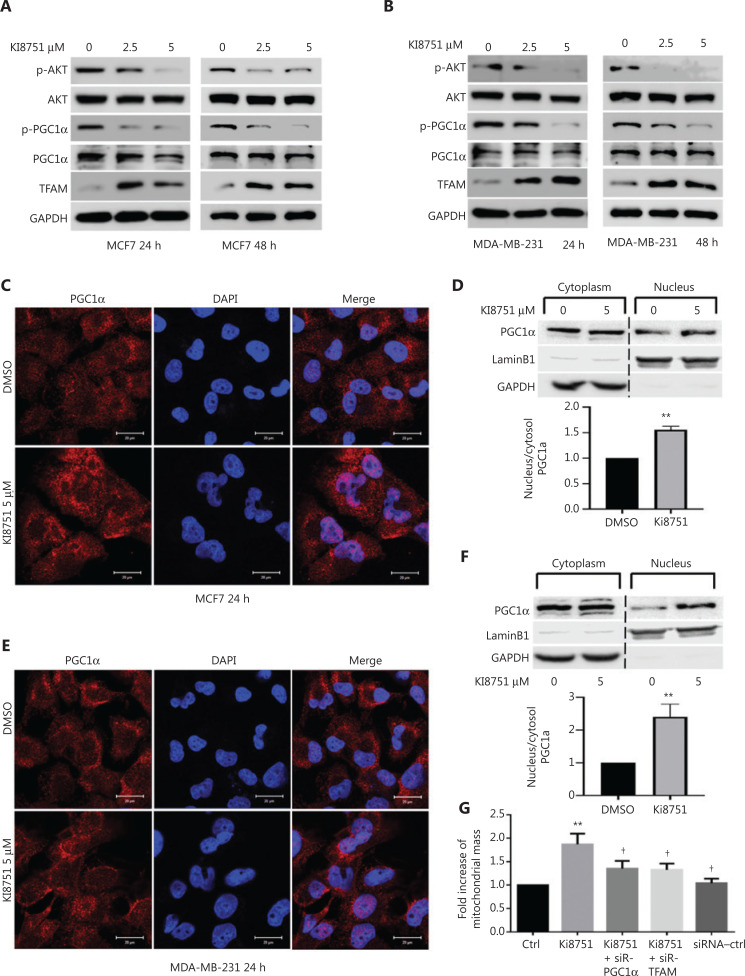
Vascular endothelial growth factor receptor (VEGFR) 2 blockade by Ki8751 interferes with VEGF intracellular signaling in breast cancer cells. The breast cancer cells, MCF-7 and MDA-MB-231, were seeded in 6-well plates (1.5 × 10^5^ cells/well), and cultured with vehicle (0.01% dimethyl sulfoxide), 2.5, or 5 μM Ki8751 for 24 or 48 h. The whole cell lysates were prepared using EBC buffer (50 mM Tris at pH8.0, 120 mM NaCl, 0.5% NP-40) containing protease inhibitors and phosphatase inhibitors. Samples containing the same protein amounts were separated by 10% SDS-PAGE and blotted onto nitrocellulose membranes. Representative blotting images of p-Akt, Akt, p-PGC1α, PGC1α, and TFAM of MCF-7 cell (panel A) and MDA-MB-231 cell lysates (B) are shown from not less than 3 independent experiments. To demonstrate PGC1α intracellular mobilization, MCF-7 cells and MDA-MB-231 cells were treated with 5 μM Ki8751 for 24 h and fixed with 4% paraformaldehyde. Representative fluorescent images of PGC1α and 4′6′-diamidino-2-phenylindole are shown in (C and E) and representative immunoblotting analysis of cytoplasmic and nuclear fractions from MCF-7 and MDA-MB-231 cells are shown in (D and F); mean ± SEM are plotted in the bar charts, *n* = 4, **P < 0.01. Panel G: MCF-7 cells were treated with vehicle or 2.5 μM Ki8751 in the presence or absence of siRNA SMARTPools specific PGC1α or mitochondrial transcription factor A for 48 h. Afterwards, cancer cells were harvested for flow cytometric analysis of mitochondrial mass. Mean ± SEM, *n* = 4, **P < 0.01 compared to the control, †*P* < 0.05, compared MCF-7 cells treated by Ki8751 alone.

To further elucidate the signaling mechanisms of VEGF blockade-enhanced mitochondrial biogenesis in cancer cells, we performed a series of Western blot experiments. We showed that VEGF blockade by Ki8751 treatment decreased Akt phosphorylation at both 24 and 48 h of culture and in both MCF-7 cells (**Figure 7A**) and MDA-MB-231 cells (**Figure 7B**). The reduced AKT phosphorylation/activity decreased its phosphorylation capacity of its downstream and mitochondrial-related signaling molecule, PGC1α. Consistent with the observations that the level of PGC1α phosphorylation was reversibly correlated to PGC1α functional activities^21,22^, the low phosphorylation of PGC1α resulted in elevated expressions of TFAM in Ki8751-treated MCF-7 and MDA-MB-231 cells.

Furthermore, confocal microscopy revealed that Ki8751 treatment induced PGC1α translocation from the cytoplasm to the nucleus both in MCF-7 (**Figure 7C)** and MDA-MB-231 cells (**Figure 7E**). The PGC1α mobilization was seen as increased PGC1α fluorescence within the nuclei. The morphological evidence was further supported by immunoblotting results showing that PGC1α immunoreactivities of the nuclear fraction of Ki8751-treated MCF-7 (**Figure 7D**) and MDA-MB-231 cells (**Figure 7F**) were much more intense than those of untreated cells.

To verify the VEGFR2-PGC1α-TFAM signaling cascade, PGC1α and TFAM knockdown of MCF-7 cells were performed using corresponding siRNAs. **Figure 7G** shows that, as expected, Ki8751 treatment (2.5 μM) significantly increased the mitochondrial mass of MCF-7 cells, but both PGC1α- and TFAM-knockdown significantly reduced the Ki8751-elevated mitochondrial mass, indicating the importance of the VEGFR2-PGC1α-TFAM signaling cascade.

## Discussion

**Figure 8:**
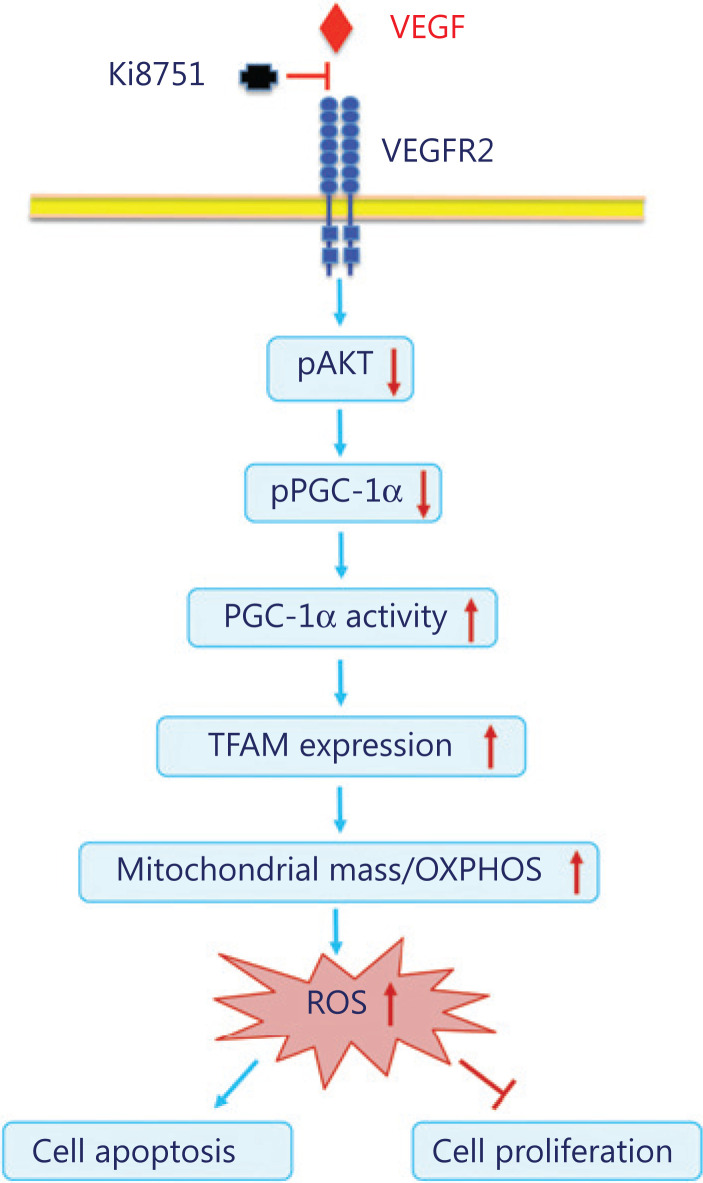
Schematic illustration of altered VEGF signaling and mitochondrial biogenesis of breast cancer cells by vascular endothelial growth factor receptor 2 blockade with Ki8751.

The present study investigated the impact of VEGF on breast cancer cell proliferation using the VEGFR2 inhibitor, Ki8751. It was found that VEGF blockade by Ki8751 inhibited cell proliferation of the breast cancer cell lines, MCF-7 and MBD-MD-231, and that the inhibition involved increased apoptosis and endomitosis of the treated cancer cells. Further results showed that VEGF blockade by Ki8751 decreased the phosphorylation and activity of Akt, and led to reduced PCG1α phosphorylation. The latter enhanced TFAM expression, and subsequently promoted mitochondrial biogenesis and ROS production that resulted in apoptosis of cancer cells (**Figure 8**).

VEGF is best known for its ability to stimulate the formation of blood vessels, in vasculogenesis (*de novo* formation of the vessels) and angiogenesis (vessel expansion of the existing vasculature)^23,24^. The VEGF family includes multiple isoforms of VEGF-A, VEGF-B, VEGF-C, VEGF-D, and VEGF-E, which bind to various VEGF receptors, i.e., VEGFR-1, VEGFR-2/KDR, and VEGFR-3. Among these receptors, VEGFR-2, also called KDR, binds VEGF-A, C, D, and E, and is highly expressed on vascular endothelial cells. VEGFR-2 signaling stimulates migration, survival, and proliferation of endothelial cells, as well as endothelial permeability^25^. VEGF-VEGFR-2 signaling impacts not only endothelial functions and angiogenesis, but also the functions of other cell types^26^. Thus, VEGF has been shown to contribute to the epithelial to mesenchymal transition and survival of breast cancer cells^27,28^. VEGFR-2 signaling has also been shown to maintain the proliferative potentials of colorectal cancer cells^10^, ovarian cancer cells^29^ as well as gastric cancer cells^30^. We have recently reported that VEGF is the key platelet-released factor in promoting proliferation of breast cancer cells^9^. To further develop our earlier work, the present study was aimed at elucidating how VEGF-VEGFR2 signaling regulates cancer cell proliferation. We confirmed our earlier findings^9^ that VEGFR2 blockade by its inhibitor, Ki8751, suppressed cancer cell proliferation. It should be noted that micromolar levels of Ki8752 were required for the inhibition of cancer cell proliferation as compared to the nanomolar levels for endothelial cell proliferation, because VEGFR2 expression on cancer cells is much lower than that on endothelial cells^26^. Moreover, the observed effects of Ki8751 were achieved specifically *via* VEGFR2 inhibition, because Ki8751 effects could be mimicked by VEGFR2 knockdown (**Figure 3H and 3I**). Using mass spectrometric analyses, we found that most elevated proteins in Ki8751-treated cancers were all mitochondrial proteins, namely NDFIP1, CLK1, and TEFM, suggesting that Ki8751 treatments may enhance mitochondrial biogenesis of breast cancer cells. Indeed, confocal microscopic observations showed that VEGFR2 blockade significantly increased mitochondrial fluorescence in both MCF-7 and MDA-MB-231 breast cancer cells, while flow cytometric analyses showed that VEGFR2 blockade elevated mitochondrial masses of both cancer cells up to 2-fold. Importantly, the present work clearly showed that enhanced mitochondrial biogenesis was associated with enhanced mitochondrial functions, seen as elevations of oxygen consumption and ROS production. The latter sequentially led to enhanced apoptosis but hampered cell proliferation of breast cancer cells. It should also been noted that VEGFR2 blockade also increased the size of the cancer cells, with larger lobular or multiple nuclei, indicating the endomitosis of Ki8751-treated cancer cells. This phenomenon was further supported by the evidence showing that Ki8751 treatment detained the cells at high aneuploid phase (G4) in cell cycle analyses, and that Ki8751 inhibition delayed M phase progression^11^.

Mitochondrial biogenesis is a subtly regulated process that requires closely coordinated expressions of nuclear and mitochondrial DNA to adapt mitochondrial mass to the energy demands of the cells, and to different physiological and microenvironmental conditions. Under the condition of increased energy demand, such as exercise, cells increase mitochondrial biogenesis to adjust their metabolic processes to maintain sufficient cellular energy to meet the energetic needs of tissues. VEGF binding to VEGFR2 leads to receptor dimerization and activation of its intracellular tyrosine kinase domains. The latter can induce Src signaling, activate PI3K, and subsequently enhance Akt phosphorylation/kinase activity^31^. The present study showed that VEGFR2 blockade by Ki8751 attenuated Akt phosphorylation, i.e., Akt kinase activity. The reduced Akt kinase activity hampered the ability of Akt to inhibit apoptotic processes^31^, and could contribute to the enhanced cancer cell apoptosis caused by Ki8751 treatment. However, the low Akt kinase activity decreased phosphorylation levels of PCG1α. Because PCG1α phosphorylation is reversely correlated to its transcriptional activity to stimulate TFAM synthesis^32^, the reduction enhanced TFAM expression. Furthermore, we have for the first time shown that VEGFR2 blockade mobilized PCG1α from the cytoplasm to the nucleus of both breast cancer cell lines, as evidenced by confocal imaging and nuclear fraction blotting of PCG1α. Hence, nuclear-translocated PGC1α binds to nuclear transcription factors to form a promoter complex that promotes TFAM expression^33^. TFAM is the critical transcription factor for mitochondrial DNA (mtDNA), and it is essential for mtDNA replication and transcription^18^. TFAM contains two HMG-box domains, which are DNA binding motifs that insert into the minor groove of DNA on the light strand promoter, heavy strand promoter 1, or nonspecific regions of the mtDNA. TFAM-binding causes a distortion of the mtDNA and leads to DNA bending. This distortion enables POLRMT (mitochondrial RNA polymerase) to bind to the initiation site, where TFAM-binding to the N-terminus of POLRMT helps to recruit another mitochondrial transcription factor , mitochondrial Transcription Factor 2 while maintaining an open DNA complex arrangement that enables productive transcription initiation^34^. Based on these complex mechanisms, TFAM facilitates mtDNA replication and transcription^19^.

Taken together, VEGFR2 blockade by Ki8751 attenuates Akt phosphorylation and activity that reduces PCG1α phosphorylation, but enhances PCG1α activity and translocation into the cell nucleus. The PCG1α alterations stimulate TFAM expression, mitochondrial mtDNA transcription, and mitochondrial biogenesis. Increased mitochondrial mass results in enhanced mitochondrial respiration and ROS production that, in turn, induces apoptosis and inhibits cell proliferation of cancer cells. Therefore, increased mitochondrial mass caused by VEGFR2 blockade may trigger the metabolic reprogramming of cancer cells from glycolysis to oxidative phosphorylation (**Figure 8**). In conclusion, VEGF-VEGFR2 signaling plays a vital role in homeostasis of mitochondrial biogenesis, to hamper apoptotic processes, and to maintain cell proliferative potential of breast cancer cells. Therefore, intervention of VEGF-VEGFR2 signaling represents a useful strategy to add to current therapeutic approaches of breast cancer.
